# Autoinflammatory Mechanisms in Crystal-Induced Arthritis

**DOI:** 10.3389/fmed.2020.00166

**Published:** 2020-04-30

**Authors:** Francesca Oliviero, Sara Bindoli, Anna Scanu, Eugen Feist, Andrea Doria, Paola Galozzi, Paolo Sfriso

**Affiliations:** ^1^Rheumatology Unit, Department of Medicine—DIMED, University of Padova, Padova, Italy; ^2^Department of Rheumatology, Cooperation Partner of the Otto-von-Guericke, University Magdeburg, Helios Clinic, Vogelsang-Gommern, Germany

**Keywords:** crystal-induced arthritis, interleukin-1, autoinflammatory disease, interleukin-1 receptor antagonist, inflammasome

## Abstract

Crystal-induced arthritides have been classified as “type-1 autoinflammatory diseases” for their main features which resemble those of the monogenic autoinflammatory syndromes. They are in fact characterized by spontaneous onset, recurrence of the episodes, self-limitation and resolution, inflammasome activation with huge production of IL-1β and a prevalent involvement of the innate immune system. The term “auto” refers also to the induction of IL-1β gene expression, processing and secretion by IL-1β itself. The concept of autoinflammation in crystal-induced arthritis has been finally reinforced by the efficacy of IL-1 blockade in treating acute and chronic state of this disease. The aim of this article is to review the autoinflammatory mechanisms in crystal-induced arthritis, considering both clinical and molecular aspects.

## Introduction

Crystal-induced arthritis (CIA) comprises two main inflammatory arthropathies, gout, and pseudogout, characterized by the formation and deposition in joints of monosodium urate (MSU) and calcium pyrophosphate (CPP) crystals, respectively. A third type of crystals, named basic calcium phosphate (BCP) crystals are associated with calcific periarthritis, osteoarthritis, and destructive arthropathies. The main features of CIA resemble those of the monogenic autoinflammatory syndromes such as: spontaneous onset, recurrence of the episodes, self-limitation and resolution, inflammasome activation with huge IL-1β production with prevalent involvement of the innate immunity (1). Although no specific genetic causes, but rather multi-genetic predispositions have been associated with CIA, gout and pseudogout have been classified as “type-1 autoinflammatory diseases” (2).

The initial observation that uric acid is able to stimulate the innate immune system dates to 2003 when it was identified as a major endogenous danger signal released from injured cells capable to activate T-cell responses (3).

Two following works established the importance of IL-1β in CIA and laid the foundation for the definition of gout as autoinflammatory disease. The first concerned the demonstration that uric acid crystals (likely together with additional triggers) were able to activate the cytoplasmic NACHT-LRRPYD-containing protein-3 (NLRP3) inflammasome which, through the caspase-1 activation, induced cleavage of pro-IL-1β to IL-1β (4). The second, which was conducted simultaneously, demonstrated the crucial role of IL-1 receptor and MyD88 molecule in non-myeloid derived cells, in triggering and amplify the inflammatory response induced by pathogenic crystals (5).

The prefix “auto” in autoinflammation does not refer only to the spontaneous resolution, but also implies that IL-1 induces its own gene expression, processing and secretion (6), and therefore, the chronic triggering of IL-1 receptor by IL-1β itself can be considered as an autoinflammatory process (7).

The efficacy of IL-1 blockade in treating acute and chronic crystal arthritis has finally reinforced the concept of autoinflammation in those diseases (8, 9).

The aim of this article is to review the autoinflammatory mechanisms in CIA, considering both the molecular and clinical aspects.

## Clinical Course

The recurrent and apparently not-provoked episodes of CIA are typical of autoinflammatory diseases. Both gout and pseudogout are characterized by an acute and a chronic phase. The first phase is characterized by recurrent acute attacks that resolve spontaneously over a period of 7 to 10 days, with asymptomatic periods between attacks.

**Figure 1:**
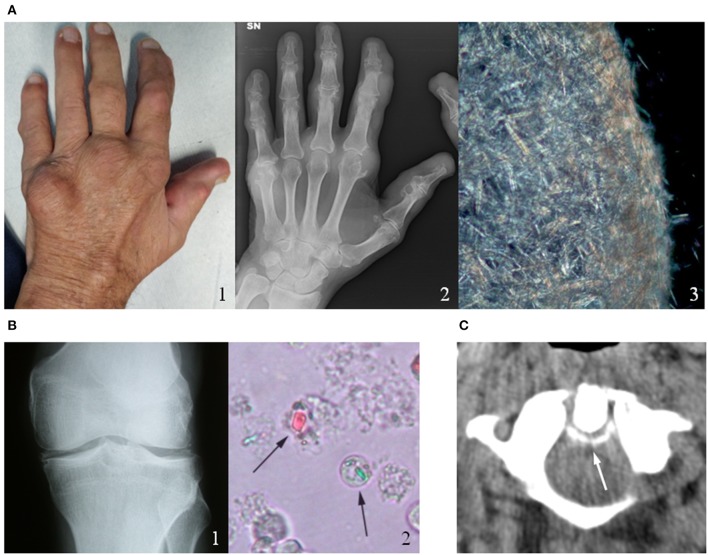
Clinical, radiological and microscopic aspects of gout and calcium pyrophosphate (CPP) crystal deposition. **(A)** Chronic tophaceous gout (1) with severe joint destructions at radiograph (2) in a male patient with long-standing polyarticular gout. Synovial fluid analysis collected from the V metacarpophalangeal joint showed an aggregate of needle-shape monosodium urate crystals (3). **(B)** CPP crystal deposition in the knee of a patient with recurrent pseudogout attacks (1). CPP crystals (arrows) observed in synovial fluid under compensated polarized light (2). **(C)** Axial computed tomography (CT) image of a male patient's cervical spine at C1-C2, illustrating the “crowning” of the dens. Arrow represents CPP deposition. Original figures obtained and reproduced with patients' written informed consent to publication.

The onset of the acute attack is abrupt, with symptoms and signs of severe acute inflammation (intense pain, redness, warmth, swelling, tenderness, and joint disability). If untreated, acute attack in gouty patients commonly resolves within a few days, while initial episodes of acute CPP crystal arthritis may persist longer (1 to 3 weeks) before remitting (10). Some patients may experience one single attack, but more often other episodes occur within 6 months to 2 years. Chronic tophaceous gout with severe joint destruction (Figure 1A) can also develop after many years of recurrent polyarticular gout. The deposition of BCP crystals leads to different clinical manifestations, the most severe of which are calcific periarthritis and the destructive arthropathy of the elderly known as Milwaukee shoulder (or knee) syndrome. While acute calcific periarthritis is characterized by a painful onset and a self-limiting course, Milwaukee shoulder syndrome is characterized by intermittent pain and large non-inflammatory effusions.

As regards affected joints, the first metatarsophalangeal joint is the most frequently affected in gout, the knee is affected in over 50 percent of all acute attacks of acute CPP crystal arthritis (Figure 1B) (11). Other joints such as the wrists, shoulders, ankles and hands can also be affected by CIA.

Gout and pseudogout may also present different extra-articular manifestations such as hypertension, obesity and dyslipidemia, which make these patients at higher risk to develop cardiovascular comorbidities and renal dysfunctions.

## Fever

The activation of NLRP3 inflammasome by crystals, which determines the final production of IL-1β, may be considered a possible mechanism for the onset of fever in patients affected by crystal arthropathies. Although systemic fever occurs in a minority of subjects with acute gout, it may be present in patients with repeated polyarticular attacks (12). As reported in literature in the past (13), fever may be more prevalent (up to 50%) in those affected by CPP crystal-induced arthritis than in gouty patients. Similar cases were reported by Berger et al. who observed the presence of fever in association with high sedimentation rate (ESR) in subjects affected by CPP deposition (14). Overall, CIA may be often the cause of febrile systemic inflammatory diseases particularly in elderly population (15). However, it is important to recognize when febrile episodes are related to infectious arthritis or a systemic sepsis. Therefore, in order to avoid misdiagnosis and uncorrected therapies, it is suggested to perform additional laboratory and microbiological tests to confirm the diagnosis. In this context, synovial fluid analysis is the most specific diagnostic test in presence of joint effusion, and it should be always performed in case of acute monoarthritis.

It was observed that in patients with chronic tophaceous gout the polyarticular repeated attacks may induce a systemic inflammatory response syndrome (SIRS) without an associated infection (16). Similarly, the uncommon axial involvement in polyarticular gout has the capability to induce a SIRS reaction-like mimicking a sepsis within the context of a chronic crystal arthropathy (17). Likewise, the crowned dens syndrome, due to calcium pyrophosphate or hydroxyapatite deposition in the cervical spine around the odontoid process (Figure 1C), may present with severe occipital pain, neck stiffness and high fever and can be often misdiagnosed as polymyalgia rheumatica or meningitis (18). In general, the prevalence of fever in crystal-induces arthropathies is driven by specific pyrogens (IL-1, IL-6, TNF-α) with the inflammasome as pivotal activator of the inflammation cascade.

## Endogenous and Exogenous Factors Involved in Triggering Crystal-Induced Inflammation

Studying the molecular mechanisms of CIA gave notable details about the NLRP3 inflammasome and the release of pro-inflammatory cytokines (4). Nevertheless, it is important to bear in mind that hyperuricemia can be asymptomatic, and crystals can be present without an inflammatory response, suggesting the existence of regulatory mechanisms that can modify the acute inflammatory response (19).

**Figure 2:**
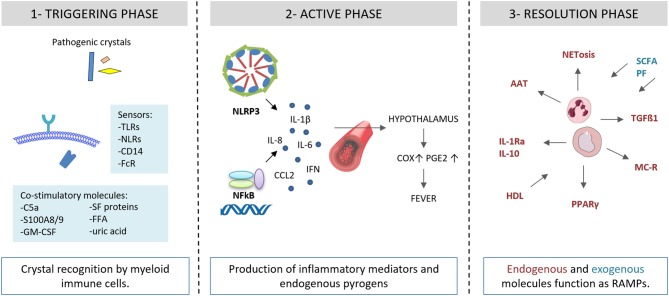
Autoinflammatory steps in crystal-induced inflammation. (1) Crystal deposition triggers the acute attack in presence of co-stimulatory molecules (C5a, S100A8/A9, GM-CSF, SF proteins, FFA, uric acid). Innate immune system receptors (TLRs, NLRs, CD14, FcR) recognize pathogenic crystals promoting acute inflammatory responses in myeloid cells (monocytes/macrophages). (2) Through NLRP3 activation, crystals induce the production of IL-1β and inflammatory mediators and endogenous pyrogens. (3) Endogenous and exogenous molecules function as RAMPs and limit the inflammatory response. AAT, alpha-1-anti-trypsin; C5a, complement factor 5a; CCL2, C-C motif chemokine ligand 2; COX, cycloxygenase; FcR, Fc receptor; GM-CSF, granulocyte-macrophage colony-stimulating factor; granulocyte HDL, high density lipoproteins; IL, interleukin; IL-1Ra, IL-1 receptor antagonist; IFN, interferon; FFA, free fatty acids; NET, neutrophil extracellular traps; MC, melanocortin; NFkB, nuclear factor kB; NLRP3, NACHT-LRRPYD-containing protein-3; NLRs, nod-like receptors, PGE, prostaglandin E; PF, polyphenols; PPARγ, peroxisome proliferator-activated receptor γ; S100A8/A9, S100 calcium-binding protein A8/A9; SCFA, short chain fatty acids; SF, synovial fluid; TGF, transforming growth factor; TLRs, Toll-like receptors.

Recent discoveries light up the complex associations of endogenous and exogenous factors at the initial step of the inflammatory inflammasome-mediated process (Figure 2). The canonical response of the inflammasome to the “second signal,” granted by the interaction of crystals with inflammasome-competent cells, requires “first signal” priming events, in order to avoid inappropriate firing of the pathway (20). The mechanisms by which MSU crystals trigger the “second signal” are still poorly understood. Perturbation in intracellular ionic homeostasis, especially potassium and calcium balance, leads to mitochondrial reactive oxygen species (ROS) generation before assembling the inflammasome (21).

Different conditions promoting an underlining inflammatory response can lead to a non specific “first signal” priming step. Endogenous factors include the complement protein C5a, the granulocyte-macrophage colony-stimulating factor (GM-CSF), the heterodimer S100A8/A9, and the protein fraction of synovial fluids. Exogenous factors include long chain saturated fatty acids and spikes in systemic levels of acetate.

New researches on exogenous activators of NLRP3 inflammasome suggest the role of a purine-rich diet, that is observed to increase 5-fold the risk of an acute attack of gout (22). The exact components of this diet are still debated, but it is known that long-chain (C18) free fatty acids (FFAs) might have a role in macrophage priming. Joosten et al. demonstrated a strong interaction of FFAs with Toll-like receptor (TLR2) that synergized with MSU crystals to induce an ASC/caspase 1–driven IL-1 release (23).

Spikes in systemic levels of a short-chain fatty acid can also contribute to the acute gout flares, as observed by Vieira et al. in a murine model of gout (24). The increase of acetate, which signals through the G protein coupled receptor GPR43, restored inflammation in response to injection of MSU crystals and, *in vitro*, leads macrophages to produce ROS and assemble the inflammasome.

Endogenous ligands of TLR4 (S100A8/A9) have a role in macrophage priming in gout. Holzinger et al. (25) observed that gouty patients and MSU-injected mice produced high level of this heterodimer and increased IL-1β secretion.

In addition, priming of monocytes of gouty patients by higher levels of soluble uric acid enhances the proinflammatory response upon subsequent exposure to TLR2 and TLR4 agonists (26). There is a concomitant downregulation of IL-1Ra that can be responsible to reinforce the enhanced state of inflammation.

An and colleagues described that C5a potentiates MSU crystals in IL-1β production in a caspase-1 dependent manner, requiring intracellular calcium mobilization, potassium efflux, and cathepsin B activity (27). P2X7R, a purinergic receptor activated by crystal-triggered ATP, is described to induces potassium efflux, ROS production and activates inflammasome signaling pathway (28).

In the study from Shaw et al. (29), GM-CSF is reported *in vitro* to differentiate monocytes into inflammatory macrophage, that can express high levels of NLRP3, active IL-1β, and active caspase-1, once stimulated with crystals.

It is also known that fibrinogen can stimulate cytokine production in macrophages through TLR4 ligation (30). Oliviero et al. (31), priming human THP-1 cells to crystals with low doses of phorbol myristate acetate, reported that fibrinogen is able to increase the inflammatory action of CPP crystals in a dose-dependent manner. Later, the same working group demonstrated that protein not lipid fraction of synovial fluid is required for the induction of IL-1β by MSU crystals in macrophages (32). In particular, proteins with molecular weight > 50 kDa, such as fibrinogen, can contribute to initiate gouty inflammation.

In this context, it is important to mention that the crystal-induced inflammation, leading to neutrophil migration and accumulation, may be also due to an inflammasome-independent pathway that ensures a strong activation of IL-1β in inflamed tissues, where neutrophils are abundant (33).

## The Role of Resolution-Associated Molecular Patterns (RAMPs)

The spontaneous resolution is one of the hallmarks of crystal-induced arthritis. Most of the factors involved in the self-limiting course of the disease have been identified as endogenous molecules that are induced or locally recruited by the inflammatory process itself or are inhibitory proteins normally present in the joint (Figure 2). Among them, transforming growth factor (TGF) β1 plays a crucial role in the resolution of crystal-induced inflammation. It has been shown to inhibit leukocyte chemotaxis (34), upregulate the cytokine inducible SH2-containing protein (CIS) (35) and transglutaminase (TG) 2 expression and intracellular negative regulators of cytokines such as the suppressors of cytokine signaling (SOCS)3 (36). Both macrophages (35) and neutrophils (37) are important sources of TGFβ1.

Similarly to TGF, the natural inhibitor of IL-1β, IL-1Ra, and IL-10 might have a key role in the resolution phase as its levels increase in SF of patients with gout (35) and following MSU injection in mice (38).

Lipoproteins have been shown to modulate crystal-induced inflammation through the inhibition of cell activation (39), IL-1β (38) and monocyte/macrophage recruitment (40).

An interesting mechanism of auto-regulation in CIA which is also associated to autoinflammatory syndromes self-resolution is NETosis. It has been observed that MSU crystals induce neutrophil cell death with the release of decondensed nuclear DNA coated with cell granule enzymes to generate neutrophil extracellular traps (NETs) (41). Although NETs have been shown to have both inflammatory and anti-inflammatory effects, NETosis has been supposed to facilitate crystal sequestration in aggregates within tissues limiting the inflammatory response. This process is driven, at least in part, by IL-1β (41).

Interestingly, Apostolidou et al. suggested that the inflammatory attacks of Familial Mediterranean Fever (FMF), could be also triggered by the IL-1β release through NETs. Conversely, NETs can serve as inhibitors of NETosis, facilitating the resolution of FMF attacks (42). These observations support a potential role for NET in crystal-induced IL-1ß production and could represents an interesting matter for further studies.

Another event which is associated with CIA resolution is the release of phosphatidylserine-rich microvescicles by infiltrating neutrophils during the inflammatory process. It has been demonstrated that these microvescicles limit inflammasome activation in C5a primed macrophages and, as a consequence, IL-1β release (43).

Different other regulatory factors involved in the spontaneous resolution of an acute attack of CIA have been described. Among them, peroxisome proliferator-activated receptor γ (PPARγ) ligands, which reduce the production of IL-1β and TNF induced *in vitro* by crystals (44) and melacortin receptor (MC-R) agonists which lower the levels of cytokines and polymorphonuclear cell migration in a murine model of MSU crystal-induced peritonitis (45).

The ketone body β-hydroxybutyrate (BHB) produced in the liver, has been shown to inhibit IL-1β processing in response to MSU crystals by blocking NLRP3 inflammasome and reducing caspase-1 activation (46).

Alpha-1-anti-trypsin (AAT), the major natural inhibitor of serine proteases produced by neutrophils has demonstrated an important inhibitory role in crystal-induced inflammation. AAT not only reduces the conversion of IL-1β precursor into the active cytokine in neutrophils but also increases circulating levels of endogenous IL-1Ra, the IL-1 natural inhibitor (47).

Other factors have recently been described for their effects in modulating the resolution of the acute attack. They are exogenous substances mainly introduced with diet which possess immune-inflammatory-regulatory properties [see review in (48)].

Among them, the most studied compounds are plant polyphenols and short-chain fatty acids.

As far as short-chain fatty acids are concerned, some interesting results have been obtained using butyrate, a major short-chain fatty acid produced during gut flora-mediated degradation of dietary fibers (49). This metabolite has been shown to suppress urate crystal-induced IL-1β production and expression through the specific inhibition of class I histone deacetylase epigenetic enzyme.

Another short-chain fatty acid, acetate, has been recently demonstrated to control the inflammatory response to MSU crystals in mice knee joint by favoring a faster resolution of the inflammatory process (50).

## Efficacy of IL-1 Inhibition in CIA

Standard treatment for CIA acute attack encompasses corticosteroids, non-steroidal anti-inflammatory drugs (NSAIDs), and colchicine. Besides its capability to block the polymerization of tubulin, colchicine is highly effective at preventing the MSU or CPP crystal-induced processing of proIL-1β and the release of IL-1β in monocytes (4) and has a direct effect on IL-1β production in neutrophils (42). Indeed, its use has been extended to a series of autoinflammatory diseases, such as FMF, Behcet's disease and idiopathic recurrent acute pericarditis.

Urate lowering therapies, including allopurinol, febuxostat, probenecid, and lesinurad, are used to prevent MSU crystals formations and gout flares. Among them, febuxostat has recently demonstrated to influence the inflammatory response decreasing IL-1ß serum levels in patients with gout (51).

A significant goal in treating patients intolerant or refractory to traditional drugs has been achieved using IL-1 inhibitors. To date, they include direct inhibitors of IL-1β (canakinumab and gevokizumab), selective inhibitors of the IL-1 receptor (anakinra) and dimeric trap fusion proteins (rilonacept).

The efficacy of anakinra in gout was firstly shown in 2007 by So et al. in a pilot study in which anakinra was successfully administered subcutaneously for 3 consecutive days in 10 patients (8). Later, the same results were observed by Chen et al. (52). Among the 10 patients enrolled, six had a complete response to the drug, three a partial one and one patient had no response. A study on a cohort of hospitalized patients for which standard therapy was ineffective or contraindicated (53) showed an improvement in acute gouty arthritis in the 73% of the subjects without the onset of notable drug-related adverse events (AEs). A broader multicenter retrospective study on anakinra was carried out in 2013 on 40 patients with an achievement of response in 90% of the subjects and the finding of AEs due predominantly to infections, appropriately treated with antibiotics. Although there are no available randomized controlled trials (RCTs) to confirm the data from the studies carried out, anakinra seems to be effective in those patients with acute gouty arthritis which do not respond to the standard therapy and for which nonsteroidal anti-inflammatory drugs (NSAIDs), corticosteroids (CS) or colchicine are contraindicated.

Few studies have instead investigated the effects of IL-1Ra in pseudogout showing that anakinra was effective and safe in two small series of patients with refractory arthritis due to acute CPP crystal deposition (9, 54, 55).

The efficacy of rilonacept in gouty arthritis attacks has been investigated in one phase 3 RCT (56) and in three RCTs for the prevention of gout flares during the uric acid lowering therapy. The results of all these studies confirmed the efficacy of the IL-1 inhibition in pain improvement and in inflammation markers decrease. However, rilonacept was not approved by EMA nor FDA for these indications so far. In contrast, canakinumab was approved by EMA and FDA in 2013 for the treatment of gout. So and colleagues in 2010 investigated in a phase 2 RCT the dose and efficacy of canakinumab in patients unresponsive to NSAIDs, colchicine or CS. Data showed a significant improvement in pain, swelling and flare recurrence in comparison to subjects treated only with CS (57). In addition, two phase 3 multicenter RCTs carried out on 456 patients (58) achieved the primary endpoint (pain score) better than the control group using CS and the risk of flares was reduced by 62% over the 12-week observation period. Of note, more AEs occurred in the canakinumab group in comparison to the control group. The most common ones were infections of the upper respiratory tract, abscesses and gastrointestinal disorders. Similarly, another study by the same authors described the usage of canakinumab in preventing acute gout flares after starting allopurinol. As a result, the number of flares was reduced by 72% in comparison to the group treated with colchicine.

## Conclusions

Gout and pseudogout are two important and disabling arthropaties with autoinflammatory features. Standard available pharmacological treatments are effective in treating the acute attack in both the diseases. However, while urate-lowering drugs are used to control uricemia and prevent recurrent attacks, no drugs capable of resolving chronic CPP-induced arthritis are currently available. Furthermore, some traditional drugs including allopurinol, corticosteroids, and colchicine are contraindicated in some patients who present drug intolerance or comorbidities such as renal dysfunctions. In those patients, an additional effective way of treatment is needed.

Over the last decade, the advanced understanding of autoinflammatory mechanisms of rare monogenic diseases led to a greater comprehension of the pathogenesis of more common multigenic rheumatic disorders, such as CIA, where innate immunity has demonstrated to play a crucial role. This had an important consequence as new treatment options have been developed for patients affected by those diseases.

Another aspect worth to be considered concerns the pro-inflammatory and damaging role of pathogenic crystals in different body districts. Indeed, cholesterol crystals are associated with vascular endothelial dysfunction and calcium phosphate crystals with vascular calcification.

The demonstration that these crystals trigger the NLRP3 inflammasome and the release of inflammatory cytokines that also drive uric acid crystal-induced inflammation indicates that a drug effective in gout might be relevant in preventing inflammation and limiting injury in other disease including atherosclerosis (59).

In the future, the characterization of new specific molecular pathways in crystal arthritides might offer additional means to treat crystal-related diseases with higher efficacy and specificity.

## Author Contributions

All authors listed have made a substantial, direct and intellectual contribution to the work, and approved it for publication.

## Conflict of Interest

The authors declare that the research was conducted in the absence of any commercial or financial relationships that could be construed as a potential conflict of interest.
